# Cambridge Shows Oxford the Way

**Published:** 1895-04-13

**Authors:** 


### The Hospital

An Institutional Journal of

The Medical Sciences and Hospital Administration, with

AN EXTRA NURSING SUPPLEMENT.

Vol. XVIII.—No. 445.] [April 13, 1895.

### CAMBRIDGE SHOWS OXFORD THE WAY.

The business of a University is to widen, purify, and strengthen civilisation and human life. Of all the "resources of civilisation" which the present century has developed, no single one has proved itself more clarifying and invigorating to the intellect, or more practically serviceable to a strenuous period than scientific medicine. A University which clearly perceives how it may best serve its generation "annexes" medical science as a matter of course. A University which looks askance at scientific medicine proves thereby that it has missed at least one of the main thought currents and practical movements of the age.

By a grace of the Senate the University of Cambridge resolved a few days ago "That the Vice-Chancellor be authorised to confer with the authorities of Addenbrooke's Hospital with a view to establish a closer and more regular connection between the hospital and the University teachers in the department of medicine." The authorities at Cambridge have had the wit to recognise what has so long been clearly seen by men of philosophic mind within medicine itself, that medicine, rightly understood, is a true and proper science of man. Neither philosophy, law, nor even religion is so fully and completely a "science of man" as the medicine of modern times. Philosophy is a specialism and deals with mind; law is a specialism, and deals with social behaviour; religion is a specialism, and deals with emotions and character. But scientific medicine takes account of them all. It is only as she observes, considers, weighs, and judges the whole man in his totality, from the cradle to the grave, that she can properly set herself to her particular practical task of ameliorating, healing, and guiding the individual and the commonwealth to higher levels of mental and bodily health and social achievements. Is such a "science of man" as this a fit and proper subject for University effort, or is it not? To ask the question is to answer it, and to answer it in the only possible way.

Oxford, tried by this standard, does not emerge honourably. She is a laggard in science; and to that extent she is a laggard in the newest developments and highest interests of the living generation. Oxford stands by the old, and in that she does well. But she also looks scornfully at the new, and in that she does ill, very ill. For after all the new, as modern medicine sees it, is not new; it is as ancient as the old, and as honourable. It is as ancient as man; it is man. For if medicine does not concern herself with man, what does she concern herself with? And if man is not old, nothing else is old, at any rate to man. The scientific knowledge of man, it is true, is new to us. But is the knowledge any the less important for that? And are we to be considered unworthy of honour for bringing ancient truths to light because our fathers failed to make great physiological discoveries? That is not logic, it is not philosophy, it is not sense; it is stupidity, and it is a stupidity in which no University, least of all Oxford, ought for a moment to allow itself to have any share.

Compare Oxford with Cambridge in regard to recent medical movements. Cambridge has a fully equipped hospital of modern excellence; Oxford has a hospital which so recently as five years ago was a disgrace to the city and the University. Cambridge is now using all possible efforts to make her hospital for practical purposes a department of the University and its teaching and life. Oxford touches her hospital with the tips of her fingers, and wonders whether she ought or ought not to have any relations whatever with it beyond those of patronage and lofty condescension. The words that spring to the mind and the pen at thought of this mental attitude shall not be written.

It is not our wish to be unceasingly holding up the conduct of Oxford in regard to medicine to the amazement of the civilised world. Nevertheless we feel an irresistible compulsion to record what has been the history of the last four or five years. In 1891 our special commissioner visited the Radcliffe Infirmary, and reported that in structure it was obsolete, in appearance it was melancholy and not even clean, in adaptability to the necessities of modern medicine it was impossible, and in general administration it was nerveless and flaccid to the last degree. In the latter part of 1894 another visit was paid, and it was then publicly recorded that many important improvements had been effected, and that so far, at any rate, as the management was concerned, there was a manifest desire and determination to bring the hospital into line with all modern requirements. But as yet that determination has not borne the wished-for fruit. The changes which have been made are good as far as they go. But they have been conceived in a spirit of pettiness, and pettiness has been the result. Oxford medicine is paralysed for lack of these things. It needs a new, or, at any rate, an entirely modernised hospital; it needs to be vitalised by the introduction of new blood and new methods into its administration; and, above all, it needs to be made an integral part of the University, and of its teaching, its life, and its work. When is all this to be done? For that it must be done sooner or later is certain. It should be done now, as it is being and has been done at Cambridge. Is it asked who is to do it? The city, the county, the University—all Oxford is to do it. Oxford stands for much in the modern world. There is a very real sense in which she stands for England. Does England, as represented by Oxford, despise the science of modern medicine? Can she afford to do so? Oxford must once more take to heart the developments and necessities of the living generation, and she must act with that large intelligence and that generous practicality which medical science and the English-speaking world have a right to expect at her hands.

